# Tetra­aqua­bis[4-(4-pyrid­yl)pyrimidine-2-sulfonato]copper(II) dihydrate

**DOI:** 10.1107/S1600536809011738

**Published:** 2009-04-02

**Authors:** Lei Li, Gang Xu, Hai-Bin Zhu

**Affiliations:** a School of Chemistry and Chemical Engineering, Southeast University, Nanjing, People’s Republic of China

## Abstract

In the title complex, [Cu(C_9_H_6_N_3_O_3_S)_2_(H_2_O)_4_]·2H_2_O, the Cu^II^ atom lies on an inversion centre and is coordinated by four water mol­ecules in equatorial positions and two N atoms from two 4-(4-pyrid­yl)pyrimidine-2-sulfonate ligands in apical positions. The asymmetric unit contains half of the complex and one free water mol­ecule. The water mol­ecules, including the uncoordinated water mol­ecules, and sulfonate O atoms are involved in O—H⋯O and O—H⋯N hydrogen-bonding inter­actions.

## Related literature

For coordination complexes with pyridine-2 sulfonate ligands, see: Kimura *et al.* (1999 ▶); Lobana *et al.* (2004 ▶). For coordination complexes with 4-(pyridin-2-yl)pyrimidine-2-sulfonate, see: Zhu *et al.* (2007 ▶).
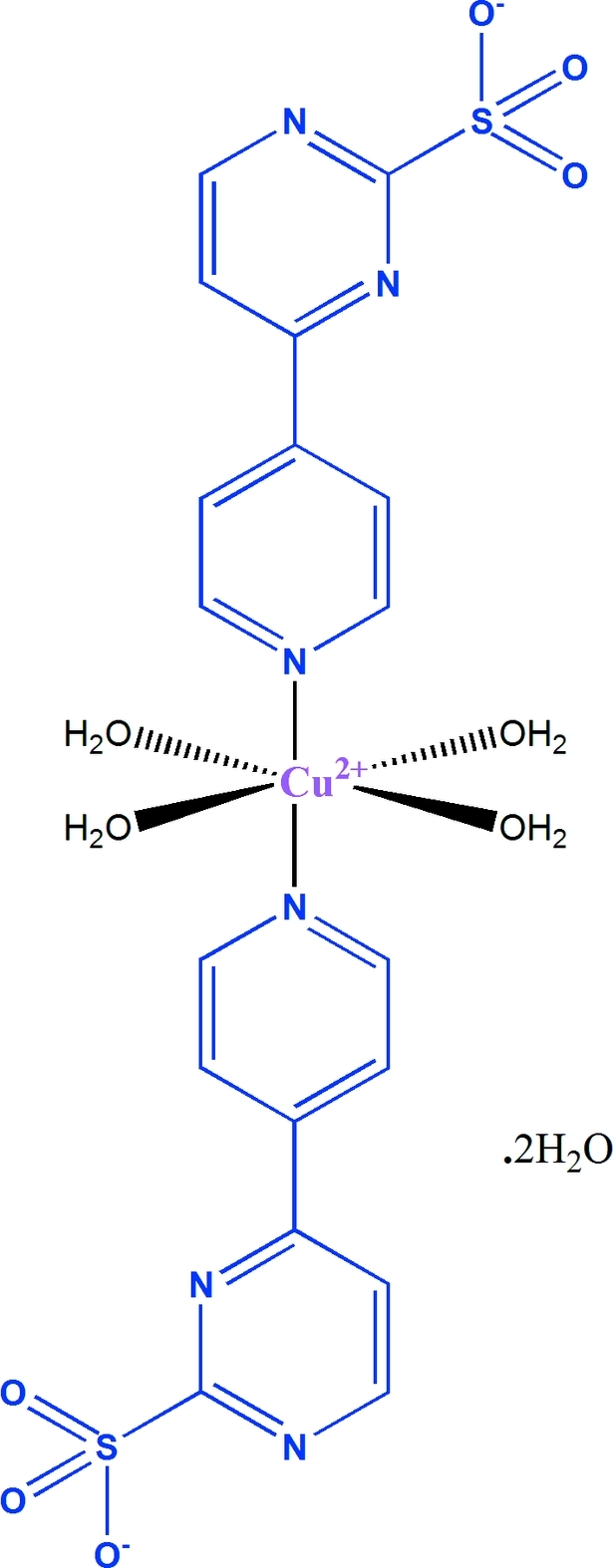


## Experimental

### 

#### Crystal data

[Cu(C_9_H_6_N_3_O_3_S)_2_(H_2_O)_4_]·2H_2_O
*M* *_r_* = 644.09
Monoclinic, 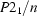

*a* = 8.0727 (11) Å
*b* = 12.1502 (16) Å
*c* = 13.4911 (17) Å
β = 95.123 (2)°
*V* = 1318.0 (3) Å^3^
*Z* = 2
Mo *K*α radiation
μ = 1.06 mm^−1^
*T* = 298 K
0.12 × 0.10 × 0.08 mm

#### Data collection

Bruker APEXII CCD area-detector diffractometer
Absorption correction: multi-scan (*SADABS*; Bruker, 2001 ▶) *T*_min_ = 0.884, *T*_max_ = 0.920
7057 measured reflections
2459 independent reflections
1786 reflections with *I* > 2σ(*I*)
*R*_int_ = 0.079

#### Refinement

*R*[*F* ^2^ > 2σ(*F* ^2^)] = 0.038
*wR*(*F* ^2^) = 0.105
*S* = 1.00
2459 reflections
197 parameters
9 restraints
H atoms treated by a mixture of independent and constrained refinement
Δρ_max_ = 0.33 e Å^−3^
Δρ_min_ = −0.37 e Å^−3^

### 

Data collection: *APEX2* (Bruker, 2007 ▶); cell refinement: *SAINT-Plus* (Bruker, 2007 ▶); data reduction: *SAINT-Plus*; program(s) used to solve structure: *SHELXS97* (Sheldrick, 2008 ▶); program(s) used to refine structure: *SHELXL97* (Sheldrick, 2008 ▶); molecular graphics: *SHELXTL* (Sheldrick, 2008 ▶); software used to prepare material for publication: *SHELXTL*.

## Supplementary Material

Crystal structure: contains datablocks I, global. DOI: 10.1107/S1600536809011738/at2756sup1.cif

Structure factors: contains datablocks I. DOI: 10.1107/S1600536809011738/at2756Isup2.hkl

Additional supplementary materials:  crystallographic information; 3D view; checkCIF report

## Figures and Tables

**Table 1:** Hydrogen-bond geometry (Å, °)

*D*—H⋯*A*	*D*—H	H⋯*A*	*D*⋯*A*	*D*—H⋯*A*
O1*W*—H2*W*⋯O2^i^	0.83 (7)	2.60 (7)	3.130 (9)	123 (7)
O1*W*—H2*W*⋯N2^i^	0.83 (7)	2.27 (4)	3.047 (10)	158 (7)
O2*W*—H4*W*⋯O3^ii^	0.82 (7)	1.91 (7)	2.734 (9)	175 (11)
O2*W*—H3*W*⋯O2^i^	0.83 (6)	1.93 (6)	2.756 (9)	169 (10)
O3*W*—H5*W*⋯O2^iii^	0.82 (6)	2.47 (7)	2.879 (10)	111 (6)

Symmetry codes: (i) 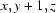; (ii) 
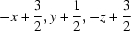
; (iii) 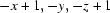.

**Fig. 1.**
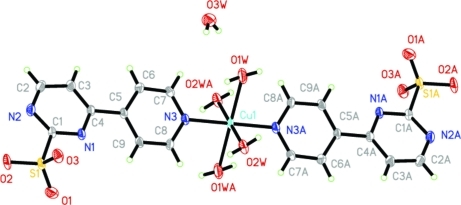
The coordination environment around Cu(II) in the title complex with the atom-labeling scheme. Displacement ellipsoids for non-hydrogen atoms are drawn at the 30% probability level.

## supplementary crystallographic information

### Comment

The coordination chemistry of some heterocyclic sulfonate ligands has been examined in several reports (Kimura *et al.*, 1999; Lobana *et al.* 2004). In our previous work (Zhu *et al.*, 2007), we have also studied divalent metal coordination complexes with the heterocyclic sulfonate ligand, namely 4-(pyridin-2-yl)pyrimidine-2-sulfonate. Herein, we report the copper(II) coordination complex with its analog, *viz* 4-(pyridin-4-yl)pyrimidine-2-sulfonate.

The coordination geometry about Cu(II) center is shown in Fig.1. The Cu(II) center adopts an octahedral coordination geometry. The equtorial plane around the copper ion is defined by four water molecules and the apical positions are occupied by two nitrogen atoms belonging to two heterocyclic sulfonate ligands. In the title complex, the Cu^II ^atom lies on an inversion centre and the asymmetric unit contains half of the complex and one free water molecule. The Cu—O bond lengths are in the range of 2.094 (6)  to 2.289 (7) Å and the Cu—N bond distance is 2.008 (7) Å. The coordinated water molecules, the guest water molecules and the free sulfonato oxgen atoms are involved in the hydrogen bonding interactions (Table 1).

### Experimental

The mixture of Cu(NO_3_)_2 _(0.1 mmol), sodium 4-(pyridin-4-yl)pyrimidine-2-sulfonate (0.2 mmol) in 6 mL of H_2_O was stirred for 20 min at room temperature. After filtration, the mother liquid was stood for three days to give the green crystals suitable for X-ray diffraction analysis.

### Refinement

All H atoms bounded to C atoms were positioned geometrically and allowed to ride on their parent atoms, with C—H = 0.93 Å. The positions of the water H atoms were found from a difference Fourier map and the positions of the water H atoms were refined isotropically by fixing the U_iso_ to 0.080.

### Crystal data

**Table:**

[Cu(C_9_H_6_N_3_O_3_S)_2_(H_2_O)_4_]·2H_2_O	*F*(000) = 662
*M**_r_* = 644.09	*D*_x_ = 1.623 Mg m^−^^3^
Monoclinic, *P*2_1_/*n*	Mo *K*α radiation, λ = 0.71073 Å
Hall symbol: -P 2yn	Cell parameters from 2459 reflections
*a* = 8.0727 (11) Å	θ = 2.3–25.5°
*b* = 12.1502 (16) Å	µ = 1.06 mm^−^^1^
*c* = 13.4911 (17) Å	*T* = 298 K
β = 95.123 (2)°	Block, blue
*V* = 1318.0 (3) Å^3^	0.12 × 0.10 × 0.08 mm
*Z* = 2	

### Data collection

**Table:**

Bruker APEXII CCD area-detector diffractometer	2459 independent reflections
Radiation source: fine-focus sealed tube	1786 reflections with *I* > 2σ(*I*)
graphite	*R*_int_ = 0.079
φ and ω scans	θ_max_ = 25.5°, θ_min_ = 2.3°
Absorption correction: multi-scan (*SADABS*; Bruker, 2001)	*h* = −9→9
*T*_min_ = 0.884, *T*_max_ = 0.920	*k* = −14→12
7057 measured reflections	*l* = −16→13

### Refinement

**Table:**

Refinement on *F*^2^	Primary atom site location: structure-invariant direct methods
Least-squares matrix: full	Secondary atom site location: difference Fourier map
*R*[*F*^2^ > 2σ(*F*^2^)] = 0.038	Hydrogen site location: inferred from neighbouring sites
*wR*(*F*^2^) = 0.105	H atoms treated by a mixture of independent and constrained refinement
*S* = 1.00	*w* = 1/[σ^2^(*F*_o_^2^) + (0.044*P*)^2^] where *P* = (*F*_o_^2^ + 2*F*_c_^2^)/3
2459 reflections	(Δ/σ)_max_ < 0.001
197 parameters	Δρ_max_ = 0.33 e Å^−^^3^
9 restraints	Δρ_min_ = −0.37 e Å^−^^3^

### Special details

**Table:**

Geometry. All e.s.d.'s (except the e.s.d. in the dihedral angle between two l.s. planes) are estimated using the full covariance matrix. The cell e.s.d.'s are taken into account individually in the estimation of e.s.d.'s in distances, angles and torsion angles; correlations between e.s.d.'s in cell parameters are only used when they are defined by crystal symmetry. An approximate (isotropic) treatment of cell e.s.d.'s is used for estimating e.s.d.'s involving l.s. planes.
Refinement. Refinement of *F*^2^ against ALL reflections. The weighted *R*-factor *wR* and goodness of fit *S* are based on *F*^2^, conventional *R*-factors *R* are based on *F*, with *F* set to zero for negative *F*^2^. The threshold expression of *F*^2^ > σ(*F*^2^) is used only for calculating *R*-factors(gt) *etc*. and is not relevant to the choice of reflections for refinement. *R*-factors based on *F*^2^ are statistically about twice as large as those based on *F*, and *R*- factors based on ALL data will be even larger.

### Fractional atomic coordinates and isotropic or equivalent isotropic displacement parameters (Å^2^)

**Table:**

	*x*	*y*	*z*	*U*_iso_*/*U*_eq_	
Cu1	1.0000	0.5000	0.5000	0.0309 (6)	
S1	0.7319 (3)	−0.21520 (17)	0.67968 (16)	0.0350 (7)	
C1	0.7066 (10)	−0.1463 (7)	0.5611 (6)	0.0296 (19)	
C2	0.6103 (12)	−0.1450 (8)	0.3997 (7)	0.044 (2)	
H2	0.5582	−0.1793	0.3436	0.053*	
C3	0.6619 (11)	−0.0369 (7)	0.3918 (7)	0.039 (2)	
H3	0.6460	0.0009	0.3318	0.047*	
C4	0.7378 (10)	0.0130 (7)	0.4763 (6)	0.030 (2)	
C5	0.8025 (10)	0.1277 (6)	0.4787 (6)	0.0285 (19)	
C6	0.7554 (11)	0.2040 (7)	0.4050 (6)	0.037 (2)	
H6	0.6823	0.1839	0.3508	0.044*	
C7	0.8170 (11)	0.3090 (7)	0.4123 (7)	0.037 (2)	
H7	0.7832	0.3590	0.3624	0.044*	
C8	0.9733 (11)	0.2692 (7)	0.5587 (6)	0.032 (2)	
H8	1.0496	0.2910	0.6107	0.038*	
C9	0.9145 (11)	0.1623 (7)	0.5569 (6)	0.033 (2)	
H9	0.9497	0.1137	0.6077	0.040*	
H1W	0.707 (11)	0.500 (5)	0.480 (5)	0.080*	
H2W	0.693 (10)	0.617 (5)	0.471 (6)	0.080*	
H3W	0.881 (8)	0.562 (5)	0.658 (7)	0.080*	
H4W	0.931 (11)	0.455 (5)	0.681 (6)	0.080*	
H5W	0.531 (10)	0.463 (9)	0.341 (4)	0.080*	
H6W	0.631 (7)	0.518 (8)	0.286 (6)	0.080*	
N1	0.7598 (8)	−0.0431 (6)	0.5630 (5)	0.0307 (17)	
N2	0.6322 (9)	−0.2021 (6)	0.4845 (5)	0.0386 (19)	
N3	0.9233 (9)	0.3430 (5)	0.4875 (5)	0.0305 (16)	
O1	0.8989 (8)	−0.1917 (6)	0.7206 (5)	0.0557 (19)	
O2	0.7032 (8)	−0.3316 (5)	0.6583 (5)	0.0497 (18)	
O3	0.6036 (8)	−0.1672 (5)	0.7337 (4)	0.0464 (17)	
O1W	0.7375 (9)	0.5611 (6)	0.4501 (6)	0.059 (2)	
O2W	0.9469 (8)	0.5109 (5)	0.6488 (5)	0.0445 (17)	
O3W	0.5402 (9)	0.4870 (7)	0.2846 (6)	0.062 (2)	

### Atomic displacement parameters (Å^2^)

**Table:**

	*U*^11^	*U*^22^	*U*^33^	*U*^12^	*U*^13^	*U*^23^
Cu1	0.0474 (10)	0.0141 (8)	0.0317 (9)	−0.0047 (7)	0.0059 (7)	0.0003 (6)
S1	0.0505 (15)	0.0172 (12)	0.0378 (14)	0.0007 (10)	0.0069 (11)	0.0024 (9)
C1	0.037 (5)	0.019 (4)	0.033 (5)	0.000 (4)	0.008 (4)	−0.003 (4)
C2	0.059 (6)	0.033 (6)	0.039 (6)	−0.009 (5)	−0.004 (5)	−0.007 (5)
C3	0.056 (6)	0.024 (5)	0.036 (5)	−0.006 (4)	−0.001 (4)	0.005 (4)
C4	0.035 (5)	0.021 (5)	0.034 (5)	0.000 (4)	0.003 (4)	−0.001 (4)
C5	0.037 (5)	0.017 (4)	0.032 (5)	−0.003 (4)	0.005 (4)	0.001 (4)
C6	0.043 (5)	0.027 (5)	0.038 (5)	−0.005 (4)	−0.006 (4)	0.002 (4)
C7	0.046 (5)	0.022 (5)	0.040 (5)	−0.001 (4)	−0.004 (4)	0.010 (4)
C8	0.044 (5)	0.021 (5)	0.030 (5)	−0.004 (4)	0.002 (4)	−0.002 (4)
C9	0.049 (5)	0.018 (4)	0.033 (5)	−0.001 (4)	0.003 (4)	0.006 (4)
N1	0.044 (4)	0.017 (4)	0.032 (4)	−0.002 (3)	0.005 (3)	0.001 (3)
N2	0.055 (5)	0.023 (4)	0.038 (4)	−0.007 (4)	0.002 (4)	0.000 (3)
N3	0.042 (4)	0.018 (4)	0.032 (4)	−0.001 (3)	0.005 (3)	0.002 (3)
O1	0.056 (4)	0.046 (5)	0.062 (4)	−0.001 (3)	−0.011 (3)	0.016 (4)
O2	0.080 (5)	0.015 (3)	0.056 (4)	−0.001 (3)	0.016 (4)	0.003 (3)
O3	0.064 (4)	0.037 (4)	0.040 (4)	0.008 (3)	0.015 (3)	−0.002 (3)
O1W	0.068 (5)	0.027 (4)	0.083 (5)	0.005 (4)	0.007 (4)	−0.012 (4)
O2W	0.061 (4)	0.024 (4)	0.050 (4)	0.003 (3)	0.016 (3)	0.006 (3)
O3W	0.059 (5)	0.058 (5)	0.067 (5)	−0.005 (4)	−0.004 (4)	0.009 (4)

### Geometric parameters (Å, °)

**Table:**

Cu1—N3	2.008 (7)	C4—N1	1.352 (10)
Cu1—N3^i^	2.008 (7)	C4—C5	1.487 (11)
Cu1—O2W	2.094 (6)	C5—C6	1.388 (11)
Cu1—O2W^i^	2.094 (6)	C5—C9	1.391 (11)
Cu1—O1W	2.289 (7)	C6—C7	1.370 (12)
Cu1—O1W^i^	2.289 (7)	C6—H6	0.9300
Cu1—H1W	2.36 (9)	C7—N3	1.335 (11)
Cu1—H3W	2.53 (7)	C7—H7	0.9300
S1—O1	1.439 (7)	C8—N3	1.349 (10)
S1—O3	1.442 (6)	C8—C9	1.382 (11)
S1—O2	1.458 (6)	C8—H8	0.9300
S1—C1	1.801 (8)	C9—H9	0.9300
C1—N1	1.324 (10)	O1W—H1W	0.89 (3)
C1—N2	1.334 (10)	O1W—H2W	0.83 (7)
C2—N2	1.336 (11)	O2W—H3W	0.83 (6)
C2—C3	1.385 (12)	O2W—H4W	0.82 (7)
C2—H2	0.9300	O3W—H5W	0.82 (6)
C3—C4	1.384 (12)	O3W—H6W	0.82 (7)
C3—H3	0.9300		
			
N3—Cu1—N3^i^	180.000 (1)	N2—C2—C3	122.7 (8)
N3—Cu1—O2W	93.0 (3)	N2—C2—H2	118.7
N3^i^—Cu1—O2W	87.0 (3)	C3—C2—H2	118.6
N3—Cu1—O2W^i^	87.0 (3)	C2—C3—C4	117.8 (8)
N3^i^—Cu1—O2W^i^	93.0 (3)	C2—C3—H3	121.1
O2W—Cu1—O2W^i^	180.000 (1)	C4—C3—H3	121.1
N3—Cu1—O1W	90.7 (3)	N1—C4—C3	120.3 (8)
N3^i^—Cu1—O1W	89.3 (3)	N1—C4—C5	115.8 (7)
O2W—Cu1—O1W	89.9 (3)	C3—C4—C5	123.9 (8)
O2W^i^—Cu1—O1W	90.1 (3)	C6—C5—C9	117.3 (8)
N3—Cu1—O1W^i^	89.3 (3)	C6—C5—C4	122.5 (8)
N3^i^—Cu1—O1W^i^	90.7 (3)	C9—C5—C4	120.2 (7)
O2W—Cu1—O1W^i^	90.1 (3)	C7—C6—C5	119.7 (8)
O2W^i^—Cu1—O1W^i^	89.9 (3)	C7—C6—H6	120.1
O1W—Cu1—O1W^i^	180.000 (1)	C5—C6—H6	120.1
N3—Cu1—H1W	72.1 (15)	N3—C7—C6	123.2 (8)
N3^i^—Cu1—H1W	107.9 (15)	N3—C7—H7	118.5
O2W—Cu1—H1W	79.5 (18)	C6—C7—H7	118.4
O2W^i^—Cu1—H1W	100.5 (18)	N3—C8—C9	122.1 (8)
O1W^i^—Cu1—H1W	158.0 (4)	N3—C8—H8	119.0
N3—Cu1—H3W	102.5 (19)	C9—C8—H8	119.0
N3^i^—Cu1—H3W	77.5 (19)	C8—C9—C5	119.8 (8)
O2W—Cu1—H3W	17.7 (12)	C8—C9—H9	120.1
O2W^i^—Cu1—H3W	162.3 (12)	C5—C9—H9	120.1
O1W—Cu1—H3W	75.0 (16)	C1—N1—C4	116.4 (7)
O1W^i^—Cu1—H3W	105.0 (16)	C1—N2—C2	114.6 (8)
H1W—Cu1—H3W	69 (3)	C8—N3—C7	117.9 (7)
O1—S1—O3	114.6 (4)	C8—N3—Cu1	120.1 (6)
O1—S1—O2	113.3 (4)	C7—N3—Cu1	122.0 (6)
O3—S1—O2	112.6 (4)	Cu1—O1W—H1W	83 (6)
O1—S1—C1	106.0 (4)	Cu1—O1W—H2W	126 (7)
O3—S1—C1	103.4 (4)	H1W—O1W—H2W	112 (4)
O2—S1—C1	105.8 (4)	Cu1—O2W—H3W	113 (6)
N1—C1—N2	128.2 (8)	Cu1—O2W—H4W	121 (7)
N1—C1—S1	114.4 (6)	H3W—O2W—H4W	114 (8)
N2—C1—S1	117.4 (6)	H5W—O3W—H6W	107 (8)

Symmetry codes: (i) −*x*+2, −*y*+1, −*z*+1.

### Hydrogen-bond geometry (Å, °)

**Table:**

*D*—H···*A*	*D*—H	H···*A*	*D*···*A*	*D*—H···*A*
O1W—H2W···O2^ii^	0.83 (7)	2.60 (7)	3.130 (9)	123 (7)
O1W—H2W···N2^ii^	0.83 (7)	2.27 (4)	3.047 (10)	158 (7)
O2W—H4W···O3^iii^	0.82 (7)	1.91 (7)	2.734 (9)	175 (11)
O2W—H3W···O2^ii^	0.83 (6)	1.93 (6)	2.756 (9)	169 (10)
O2W—H3W···S1^ii^	0.83 (6)	2.99 (4)	3.794 (7)	163 (7)
O3W—H5W···O2^iv^	0.82 (6)	2.47 (7)	2.879 (10)	111 (6)

Symmetry codes: (ii) *x*, *y*+1, *z*; (iii) −*x*+3/2, *y*+1/2, −*z*+3/2; (iv) −*x*+1, −*y*, −*z*+1.
